# A Rare Presentation of Fournier’s Gangrene: Necrotizing Infection Traveling Through a Fistula From the Rectum to the Corpus Cavernosum

**DOI:** 10.7759/cureus.89811

**Published:** 2025-08-11

**Authors:** Donald Dennis, Michael Gentry

**Affiliations:** 1 Radiology, Arrowhead Regional Medical Center, Colton, USA

**Keywords:** abdominal radiology, colorectal adenocarcinoma, corpus cavernosa, ct imaging, emergency and trauma radiology, fournier gangrene, necrotizing fascitis, pneumo peritoneum, rectal fistula, urology emergency

## Abstract

A corpus cavernosum filled with air is a rare finding during imaging, and it should raise suspicion for a serious urologic injury or infection. We present the case of a patient with air in their corpus cavernosum seen on emergency computed tomography. He was diagnosed with Fournier’s gangrene, secondary to an infectious spread through a fistula from the rectum to the corpus cavernosum. Radiologists and clinicians should consider rare presentations of necrotizing infections when encountering an air-filled corpus cavernosum on imaging.

## Introduction

Air within the corpus cavernosum is a rare finding on computed tomography (CT) that indicates a serious urologic injury or disease process [1]. The differential for air within the corpus cavernosum includes traumatic injury to the penis or urethra, ischemia from vascular disease such as priapism, rare presentations of gonorrhea, and gas-forming infection [2]. Another infrequent presentation would involve a fistula tract from the rectum to the corpus cavernosum [3]. There is a scarcity of literature reporting cases of air in the corpus cavernosum in the absence of cellulitis and subcutaneous emphysema of the penis. We present a rare case of air in the corpus cavernosum with multiple possible etiologies, including a rectal-cavernous fistula, necrotizing infection of the perineum (Fournier’s gangrene), and recent instrumentation (Foley catheter insertion) before the CT imaging of the pelvis.

## Case presentation

We present the case of a 75-year-old male patient with a past medical history of metastatic rectal adenocarcinoma who presented to the emergency department with three days of altered mental status and bloody urine. He had been hospitalized three weeks prior with rectal abscesses and colitis for which he underwent a course of metronidazole. Upon presentation to the emergency department, he was tachycardic with a heart rate of 120 beats per minute (reference range: 60-100 beats per minute) and had leukocytosis with a white blood cell count of 15,000 cells per microliter (reference range: 4500-11000 cells per microliter). A Foley catheter was placed on arrival. He had mild pain upon palpation of the penis during insertion, but there was no crepitus, erythema, or signs of infection of the penis or perineum, such as gangrenous wounds. The Foley catheter drained urine when placed into the bladder, demonstrating that a traumatic insertion or transection through the urethra was unlikely. He underwent CT imaging of the abdomen and pelvis for abdominal distension. 

**Figure 1:**
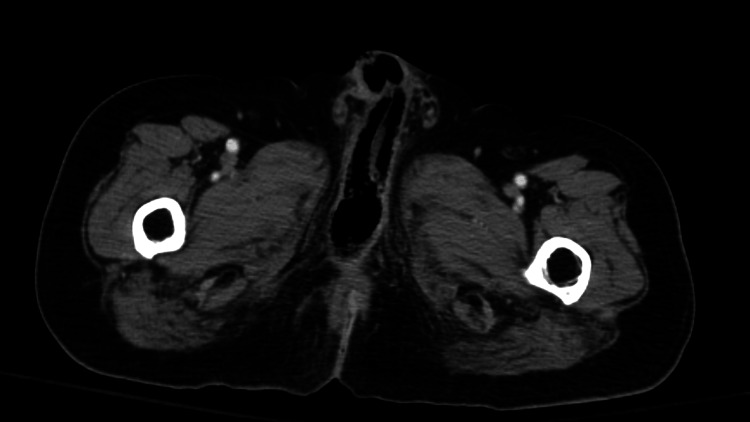
Contrast-enhanced CT axial image of the pelvis demonstrated air within the corpus cavernosum There were inflammatory changes in the fat and soft tissues of the perineum.

CT of the abdomen and pelvis demonstrated diffuse air within the corpus cavernosum of the penis with minimal air in the subcutaneous tissues of the penis (Figure 1).

**Figure 2:**
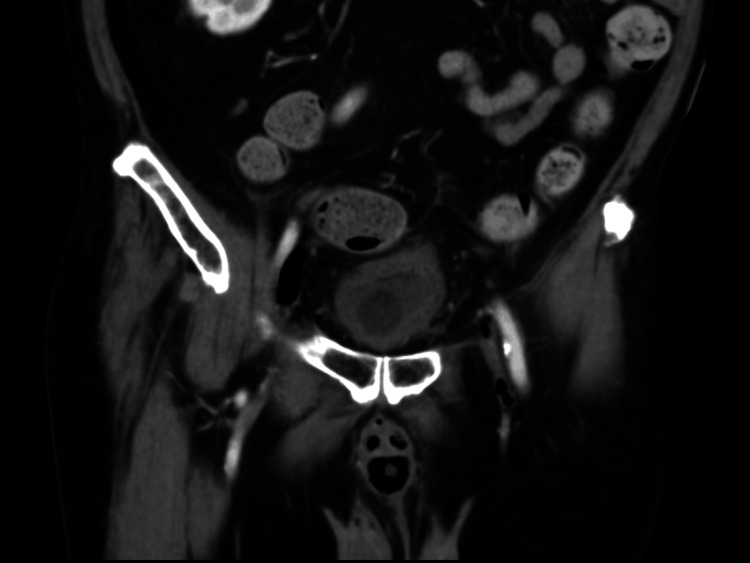
Contrast-enhanced CT coronal reconstruction image of the pelvis The image demonstrated bladder wall thickening, air in the corpus cavernosum/corpus spongiosum, air along the fascial planes of the pelvis, and small foci of air in the left common femoral vein.

There were foci of air along the perineum and within the peritoneum. The bladder was contracted with a thickened wall (Figure 2).

**Figure 3:**
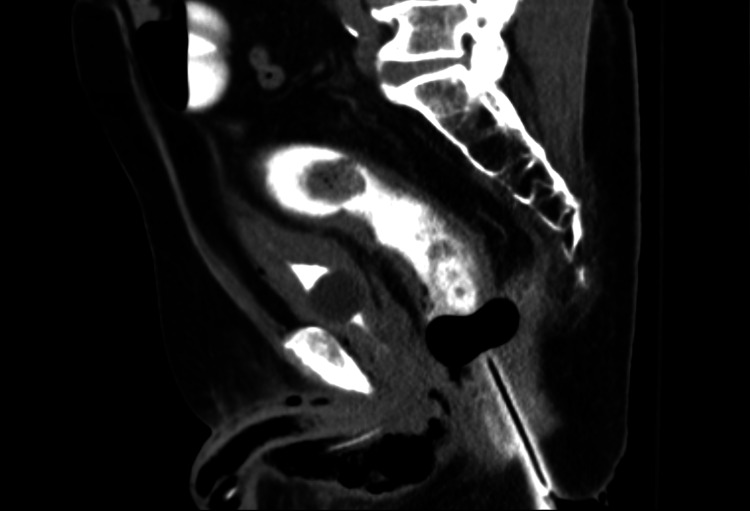
Sagittal reconstruction CT of the pelvis, enhanced by intravenous (IV) and rectal contrast Rectal contrast is seen proximal to the inflated balloon of the rectal tube. There was air in the rectal wall and along a tract in the soft tissues of the perineum to the air-filled corpus cavernosum. There were scattered foci of air along the bladder wall. Of note, the contrast within the bladder was likely excreted IV contrast from prior scans.

The patient was diagnosed with a necrotizing infection of the rectum, perineum, and penis. He was evaluated by the general surgery and urology services, who ordered a repeat CT of the abdomen and pelvis with rectal contrast. The follow-up imaging revealed an air-filled tract from the rectum communicating with the corpus cavernosum (Figure 3).

Combining findings from the clinical exam and imaging, a diagnosis of Fournier's gangrene was made. Bacterial cultures from the blood were positive for bacteremia from *Streptococcus constellatus* and *Bacteroides fragilis*. After an extensive goals of care discussion with the surgical teams, oncology team, and palliative medicine team, the patient and his family elected to forego surgical intervention and limit treatment to intravenous (IV) antibiotics and palliative care. He was eventually discharged home to continue antibiotics and palliative care in a relatively stable condition, with persistent leukocytosis, but without tachycardia or fever.

## Discussion

Fournier’s gangrene is a necrotizing infection of the perineum that leads to rapid tissue destruction, sepsis, and a high mortality rate (approximately 40% worldwide) [1]. The diagnosis is made clinically, and imaging may assist in making the diagnosis, but imaging alone cannot rule out Fournier's gangrene [1,4]. The typical imaging findings include soft tissue inflammation, abscess formation, asymmetric fascial thickening, abnormal fluid collections, and subcutaneous emphysema in the perineum or scrotum [4]. The infection spreads along the fascial planes of the pelvis, including the Scarpa's, Camper's, Colles', and Dartos fascia [1,4]. The initial diagnosis of Fournier’s gangrene was not made clinically before the imaging of our patient. Typical physical exam findings were not present, as described in the case presentation. While the patient did not present with a physical exam that was classic for Fournier’s gangrene, his clinical picture was one of a necrotizing infection of the perineum. The presence of gas on imaging, not only in the corpus cavernosum, but within the rectal abscesses and the soft tissues of the perineum, suggested bacterial infection [5], which was later confirmed by the blood cultures. As stated above, Fournier’s gangrene typically spares the corpus cavernosum and the penis, so this case would either be a rare presentation of Fournier’s gangrene or the presence of another pathologic process [6].

Repeat CT imaging of the abdomen and pelvis, but with rectal contrast, was performed to evaluate for fistula formation. The patient’s history of rectal adenocarcinoma, along with prior colitis and perirectal abscesses, both increased the pretest probability of identifying a rectal perforation or fistula [7]. In a positive CT imaging study, the rectal contrast would be seen extravasating out of the bowel lumen or appearing in a linear pattern, traveling from the bowel lumen through a fistula [8]. Neither of these imaging findings was seen on this patient’s repeat scan. Instead, there was a break in the rectal wall with gas that communicated through a tract along the perineum (Figure 3). There was a contiguous tract of gas from the rectum to the air-filled corpus cavernosum. This demonstrated the route and mechanism by which the corpus cavernosum became filled with air. The reason there was no contrast through the tract or in the penis was that the rectal tube balloon, which kept the tube in a stable position, was positioned proximal to the fistula. Hence, the contrast exiting the tube could not travel past it into the rectal opening of the tract. The tract could have been created due to the adenocarcinoma or infection [7,8]. 

In many cases, Fournier’s gangrene is managed surgically with extensive debridement and removal of the infected tissue [2]. During this exploration and debridement, the surgeon may have been able to examine the rectum and perineum to visibly see the fistula tract, and confirm its connection between the rectum and the corpus cavernosum [9]. Magnetic resonance imaging of the rectum could also have demonstrated the path of the fistula [10]. Neither of these was performed per the patient’s and family’s wishes, and we respected their autonomy and decisions for care. If the patient and their family had decided on aggressive care, further investigation into the connection could have helped determine the extent of the infection and could have guided management in preventing further infections.

## Conclusions

CT imaging assists in the diagnosis of Fournier’s gangrene when classic physical exam findings are not apparent. This case illustrates the value of a multidisciplinary approach to diagnosis involving collaboration between emergency medicine physicians, radiologists, and surgeons. Air or gas in the corpus cavernosum is an imaging finding that suggests a urologic emergency. Understanding the potential etiologies of air in the corpus cavernosum and the role of imaging in the diagnostic process can help clinicians make timely and informed decisions during these emergency presentations. Furthermore, recognizing the multiple mechanisms by which air is introduced may broaden the differential to include rare cases, such as this instance in which a necrotizing infection traveled along a fistula tract from the rectum to the corpus cavernosum. 
